# MFSD2A in health and disease: lysolipid transport, barrier physiology, and translational boundaries

**DOI:** 10.1186/s43556-026-00585-3

**Published:** 2026-09-29

**Authors:** Jinheng Gan, Yang Chen, Lin Xin

**Affiliations:** Department of General Surgery, The Second Affiliated Hospital of Nanchang University, Jiangxi Province, Nanchang, 330006 China

**Keywords:** MFSD2A, Lysolipid transport, Lysophosphatidylcholine, LPC-DHA, Blood–brain barrier, Neurodevelopment

## Abstract

Major facilitator superfamily domain-containing protein 2A (MFSD2A) is a sodium-dependent lysolipid transporter that delivers lysophosphatidylcholine-bound fatty acids, particularly docosahexaenoic acid, across specialized cellular interfaces. This Review synthesizes structural, biochemical, physiological, genetic, and disease evidence through an evidence-weighted transport-to-barrier-to-disease framework. We first examine substrate recognition, sodium coupling, conformational transport, and the membrane lipid consequences of lysophosphatidylcholine uptake. We then detail the best-established physiological setting, central nervous system endothelium, where MFSD2A supports brain docosahexaenoic acid accretion, blood–brain barrier maturation, and suppression of caveolae-mediated transcytosis. Evidence from the retina, placenta, lung, kidney, epidermis, and immune cells is compared to define tissue-specific deployment rather than a universal downstream pathway. Rare biallelic variants and experimental loss-of-function models provide the strongest disease evidence, linking impaired lysolipid transport to deficient brain lipid supply, microcephaly, and neurodevelopmental abnormalities. Acquired vascular injury studies associate MFSD2A dysregulation with barrier leakage, whereas proposed roles in cancer, immunotherapy response, and patient stratification remain context-dependent and largely associative. We also assess translational concepts, including barrier stabilization, central nervous system drug delivery, biomarker development, and structure-guided modulation, emphasizing unresolved requirements for selectivity, reversibility, safety, and prospective validation. By distinguishing transporter-intrinsic mechanisms from secondary lipid remodeling and disease correlations, this Review clarifies which functions are established, which are tissue restricted, and which remain hypotheses. This framework identifies experimental priorities for linking MFSD2A activity to clinically informative phenotypes without extrapolating beyond current evidence.

## Introduction

Major facilitator superfamily domain-containing protein 2 A (MFSD2A) is best understood as a sodium-dependent lysolipid transporter whose biological consequences are shaped by tissue context [1–3]. The central issue is not whether MFSD2A participates in diverse physiological and disease settings, but how transport of lysophosphatidylcholine (LPC) species, including LPC-DHA, is translated into barrier function, neurodevelopment, and selected pathological phenotypes. Framing the field in this way broadens the discussion beyond its earlier tumor-centered emphasis while imposing a stricter evidentiary order: transport biology and barrier physiology precede oncology, drug delivery, and therapeutic inference. Accordingly, the strongest evidence concerns transporter function and barrier specialization, whereas tumor and immunotherapy findings remain downstream and tumor-type specific [4]. The resulting framework follows a transport-to-barrier-to-disease axis. At the molecular level, MFSD2A mediates sodium-dependent transport of LPC-linked fatty acyl substrates [5]. In central nervous system (CNS) endothelium, this activity is coupled to a specialized membrane lipid state, restricted caveolar trafficking, and maturation of the blood–brain barrier (BBB) [6–8]. Disease relevance is most firmly anchored by rare neurodevelopmental syndromes and experimental models in which transporter activity, DHA accretion, brain growth, or barrier stability can be measured directly [9, 10]. Evidence from the retina, placenta, alveolar epithelium, epidermis, kidney, and immune cells expands this physiological scope, but does not imply that these tissues reproduce BBB biology.

Claims are weighted according to their proximity to MFSD2A function. Direct measurements of transport, substrate uptake, tissue lipid composition, barrier phenotype, human genetics, and experimentally reproduced disease features form the mechanistic core. Context-limited or associative observations are used to define uncertainty rather than to complete an untested causal chain. These evidentiary limits are particularly important for proposed links to ferroptosis, ACSL4/LPCAT3 signaling, cancer-cell lipid rafts, pan-cancer immunotherapy prediction, drug delivery, or combined dietary PUFA and MFSD2A activation, none of which is currently established as a general MFSD2A mechanism or therapeutic strategy [11]. This evidentiary hierarchy also determines the organization of the Review. The health-and-disease perspective is intentionally asymmetric. Physiological evidence is centered on lysolipid transport, barrier specialization, and tissue lipid handling, whereas disease evidence is strongest when transporter disruption or barrier instability is linked directly to phenotype [2, 6, 10, 12]. This hierarchy allows the field to extend beyond cancer without becoming a catalog of loosely connected associations. Accordingly, MFSD2A is treated as a transporter with context-dependent disease relevance, rather than as a disease marker in search of a single downstream pathway [13, 14]. Such a framework accommodates structural biology, endothelial physiology, rare genetics, acquired vascular stress, and selected tumor observations without erasing their different levels of causal support [11, 14–16].

Within this framework, ‘health’ denotes physiological settings in which MFSD2A-dependent lysolipid transport contributes directly to barrier or tissue lipid homeostasis [2, 17–19]. ‘Disease’ refers to settings in which genetic disruption, altered barrier state, or tumor-associated expression has been documented with sufficient specificity to warrant discussion, but not generalization [9, 10, 20]. Evidence is read in descending order of certainty: established transporter and barrier mechanisms, strong but rare neurodevelopmental disease associations, and more tentative translational or oncological observations. The biological outcome of MFSD2A can therefore be organized around four interacting variables: tissue and cellular localization, substrate identity and abundance, downstream lipid utilization, and disease stage. This model replaces a binary protective-versus-pathogenic label with testable, context-specific determinants.

The Review is organized accordingly. Section "Transport biology of MFSD2A" defines MFSD2A structure, substrate selectivity, regulatory endpoints, and mechanistic boundaries. Section "MFSD2A in health: barrier physiology and lipid homeostasis" examines blood–brain barrier physiology, endothelial lipid homeostasis, and tissue-specific transport beyond the central nervous system. Section "MFSD2A in inherited and acquired disease" evaluates inherited and acquired disease evidence, from neurodevelopmental disorders and barrier injury to restricted ocular, inflammatory, metabolic, and tumor contexts. Section "Translational opportunities and therapeutic constraints" assesses barrier modulation, drug delivery, human biomarkers, patient stratification, and drug-discovery tools under explicit translational constraints. Section "Controversies and future perspectives" integrates the principal controversies and defines priorities for causal, safety, and validation studies.

## Transport biology of MFSD2A

Any mechanistic account of MFSD2A must begin with its established identity as a sodium-dependent lysolipid transporter. This is more than a historical designation: it sets the lower boundary for interpreting subsequent observations in barrier biology, development, and disease. Structural and biochemical studies place MFSD2A within the major facilitator superfamily and demonstrate transport of LPC-bound fatty acids, with LPC-DHA as the best-characterized substrate [1, 2, 21–23]. Downstream lipid remodeling, disease associations, and therapeutic hypotheses should be considered direct consequences of MFSD2A only when transport has been linked to the relevant tissue and pathway.

### Structural basis of MFSD2A-mediated lysolipid transport

Like other secondary transporters, MFSD2A couples substrate recognition and ion binding to alternating access across the membrane [24]. Structural studies define a major facilitator superfamily architecture adapted to the translocation of LPC-DHA and related lysolipids [1, 21, 25, 26]. Its 12 transmembrane helices, sodium-binding region, and LPC-binding cavity are not simply topological features; together they constrain substrate specificity, ion dependence, and the conformational cycle through which transport occurs.

These structural features generate testable predictions. Variants or environmental perturbations that alter substrate access, sodium coupling, membrane insertion, or conformational cycling should first be expected to change lysolipid transport [1, 21, 22, 27]. By contrast, altered MFSD2A abundance in diseased tissue may represent a cause, a consequence, or a correlate of stress. Structural and biochemical measurements thus provide an essential causal filter: whenever possible, expression changes should be interpreted alongside transport activity rather than in isolation.

**Fig. 1:**
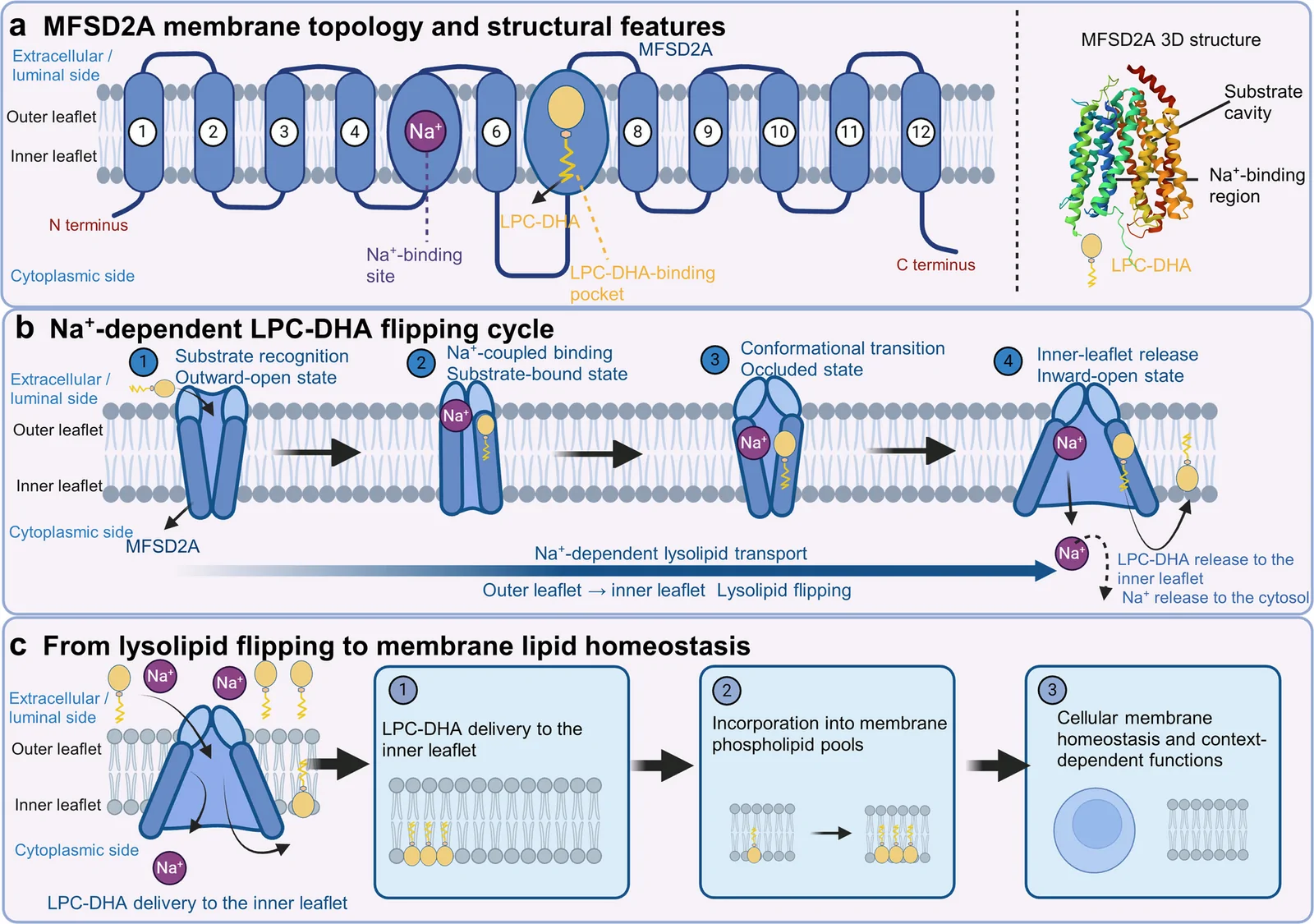
Structural basis and Na⁺-dependent lysolipid transport by MFSD2A. **a** Membrane topology and structural features of MFSD2A. The transporter comprises 12 transmembrane helices, with both termini facing the cytoplasm. The indicated Na⁺-binding region and LPC-DHA-binding cavity illustrate the structural basis of coupled ion and lysolipid recognition; the three-dimensional view highlights the central substrate cavity. **b** Simplified alternating-access cycle. Na⁺ and LPC-DHA bind to an outward-open conformation, followed by substrate occlusion and transition to an inward-open state, from which LPC-DHA is released into the inner membrane leaflet and Na⁺ into the cytosol. **c** Consequences of lysolipid flipping. Delivery of LPC-DHA to the inner leaflet enables incorporation into membrane phospholipids and thereby contributes to local lipid homeostasis. The scheme is conceptual and does not imply that all MFSD2A-dependent physiological effects converge on an identical downstream pathway. LPC-DHA, lysophosphatidylcholine carrying docosahexaenoic acid

Complete resolution of every intermediate state is not required to use the structural evidence rigorously. What matters is that sodium coordination, occupation of the substrate pocket, and conformational exchange provide a physical basis for selectivity and for the functional effects of variants or regulatory perturbations [21, 22, 25, 28]. Keeping this architecture in view separates transporter-intrinsic defects from the tissue-level responses that follow them. The structural and transport cycle is summarized in Fig. 1.

Biochemical studies establish sodium-dependent lysolipid translocation as a defining property of MFSD2A [29]. In reconstituted and cell-based assays, Mfsd2a moves lysolipid substrates from the outer to the inner membrane leaflet, and fluorescence-based experiments support a flippase-like mechanism [30]. Disruption of the sodium gradient reduces activity, further supporting ion-substrate coupling [22]. These data distinguish active transport from passive lipid association. They do not, however, substitute for tissue-specific evidence when barrier function, development, or disease phenotype is under consideration.

A transport-centered interpretation also prevents unsupported pathway assignment. Changes in lipid availability or membrane composition can influence many cellular processes without making the transporter a direct regulator of each one [31]. For MFSD2A, sodium-dependent lysolipid transport is established; proposed effects on lipid-peroxidation susceptibility, ACSL4/LPCAT3 coupling, cancer-cell lipid rafts, or antitumor immunity require separate causal evidence [21, 22, 25]. MFSD2A may condition downstream biology, but it should not be portrayed as a universal lipid-signaling switch.

### Substrate selectivity and sodium-dependent LPC transport

MFSD2A transports DHA and other fatty acids primarily when they are esterified to LPC [32, 33]. Mfsd2a-deficient mice exhibit markedly reduced brain uptake of LPC-DHA and other LPC species, together with lower brain DHA. Conversely, elevated plasma LPC species in knockout animals and individuals carrying MFSD2A mutations are consistent with impaired tissue uptake [2, 23]. These findings establish substrate selectivity at the level of LPC transport and tissue lipid availability, particularly at the blood–brain and blood-retinal barriers. Reduced delivery of LPC-linked unsaturated fatty acids may alter the lipid environment of developing and mature tissues [34].

This transport function is not confined to the CNS [32, 33]. Activated T cells lacking MFSD2A show reduced uptake of fluorescent LPC, indicating a role in immune-cell lipid acquisition [3]. In alveolar type II cells, apically localized MFSD2A recovers LPC from the alveolar surface and contributes to surfactant lipid composition [35, 36]. In the kidney, basolateral expression in S3 proximal-tubule cells, in vivo probe transport, and changes in DHA-containing phospholipids support a local transport function [37]. Differentiated epidermal cells provide another setting in which plasma-derived LPC contributes to phospholipid pools through MFSD2A [23]. The physiological outputs differ, but LPC-linked substrate handling remains the common denominator.

Substrate selectivity gives this tissue breadth a coherent mechanistic basis [32, 33]. Although LPC-DHA is the canonical cargo, MFSD2A can accommodate other LPC-bound fatty acids, with consequences that depend on local phospholipid use [2, 23]. Brain DHA accretion, retinal lipid supply, pulmonary surfactant composition, renal phospholipid pools, and immune-cell LPC uptake fit within a shared transport framework [3, 35, 37]. Their endpoints are not interchangeable; each reflects how imported lysolipids are used within a specific cellular and metabolic environment.

Tissue distribution should not be mistaken for pathway uniformity. Brain endothelium, activated T cells, alveolar type II cells, proximal-tubule cells, and epidermal keratinocytes deploy MFSD2A in distinct physiological settings [2, 3, 23, 35–37]. In endothelium, transport contributes to a membrane state that restrains vesicular traffic; in lung or immune cells, it supports surfactant composition or cellular lipid supply. The unifying feature is sodium-dependent lysolipid uptake, not a single downstream disease program [18, 38]. A broad physiological scope is therefore compatible with a narrow mechanistic core and does not recast MFSD2A as a generic cancer target or biomarker.

### Mechanistic boundaries linking transport biology to physiology and disease

Transport biology provides both the explanatory strength and the natural limit of the MFSD2A field [39]. A defined molecular mechanism can be traced to endothelial lipid state, brain DHA uptake, tissue phospholipid composition, neurodevelopmental phenotypes, and selected disease models [2, 21–23, 37]. Yet transport alone does not establish every downstream event involving fatty acids, vesicles, immune cells, or membrane signaling. A direct mechanism requires evidence that links MFSD2A activity to substrate movement, a measurable cellular or tissue lipid change, and the phenotype under study [40]. Where one or more of these links is missing, the relationship should remain associative or hypothetical.

This evidentiary boundary becomes critical once disease or therapeutic language is introduced. MFSD2A-mediated LPC-DHA transport is linked to lipid availability and barrier phenotypes in defined experimental systems [2, 3, 21–23]. Current data do not establish MFSD2A as a determinant of ferroptosis, a direct input into the ACSL4/LPCAT3 axis, a regulator of cancer-cell lipid rafts, or a validated route for sensitizing tumors through dietary PUFA and pharmacological activation. Such propositions remain questions for direct experimental testing [41]. Accordingly, the sections below carry MFSD2A forward primarily as a sodium-dependent lysolipid transporter whose consequences depend on cell type, tissue, and evidence strength [42].

Mechanistic terminology is used accordingly. ‘Direct’ is reserved for studies that connect MFSD2A activity or loss to a measured substrate, lipid state, cellular process, or phenotype [2, 10, 22]. ‘Indirect’ describes settings in which expression or abundance covaries with a process but the causal chain remains incomplete, whereas ‘hypothetical’ denotes plausible biology that has not been tested in the relevant system [43]. These categories are substantive, not semantic: they prevent a broad review from converting association into mechanism.

The same hierarchy applies to variants and experimental perturbations. A disease-causing allele, a permeability phenotype in an animal model, and an intervention that changes MFSD2A abundance provide different forms of evidence. Interpretation is strongest when transport activity, LPC-linked substrates, tissue lipid composition, and the downstream phenotype are measured within the same causal sequence [9, 10, 21, 22]. This approach allows genetic, biochemical, endothelial, and disease studies to inform one another without flattening their evidentiary differences.

### Regulation of MFSD2A expression and activity

Three regulatory endpoints must be kept separate: transcript abundance, delivery or retention of MFSD2A at the plasma membrane, and sodium-coupled lysolipid transport activity. A change in bulk RNA or immunostaining can mark an altered cellular state without demonstrating functional LPC flux, while preserved total abundance does not exclude defective trafficking, substrate access, or coupling. Disease studies should therefore not infer transporter function from expression alone.

MFSD2A regulation is best resolved as a hierarchy rather than a single upstream pathway. In brain endothelium, developmental maturation, astrocyte- and pericyte-derived signals, extracellular-matrix support, and Wnt/β-catenin activity help establish or maintain a low-transcytosis endothelial state. Cross-species and culture proteomics further show that MFSD2A abundance in isolated or stem-cell-derived models does not automatically reproduce native microvessels. Specific examples include SOX18-dependent endothelial maturation, pericyte-derived PDGF-BB/PDGFRβ signaling, and Wnt/β-catenin-associated barrier differentiation [44–60]. These observations support coordinated endothelial-state regulation, but they do not identify one universal transcriptional switch.

Placental biology provides a separate regulatory context. MFSD2A acts as a receptor for syncytin-2 at the maternal–fetal interface, and structural work together with recent in vitro reconstitution supports a physical and functional coupling between the two proteins [28, 61, 62]. In the placenta, GCM1-dependent control of MFSD2A expression provides a distinct transcriptional example [61, 63]. This receptor function should not be conflated with the regulation of LPC transport abundance or activity. Likewise, disease-associated changes in MFSD2A expression may reflect transcription, protein stability, membrane localization, substrate supply, or loss of cell identity; measuring total transcript alone cannot distinguish these possibilities.

Tumor, hypoxic, inflammatory, metabolic, and vascular settings add further heterogeneity. Promoter variation and tumor-associated expression changes have been reported, but direct transcription-factor occupancy, epigenetic causality, miRNA dependence, and post-translational control remain incompletely mapped across tissues [4, 11, 20, 64, 65]. Future studies should therefore pair expression with membrane localization, transport assays, lipidomic readouts, and perturbation of the proposed regulator. A unified upstream network is not yet established.

## MFSD2A in health: barrier physiology and lipid homeostasis

This section moves from the best-established physiological setting, central nervous system barrier endothelium, to tissue-specific functions beyond the blood–brain barrier. It first examines suppression of transcytosis and endothelial lipid homeostasis, then compares how a shared lysolipid-transport mechanism is deployed in the retina, placenta, lung, kidney, epidermis, and immune cells.

### Suppression of transcytosis and barrier maturation

The clearest physiological role of MFSD2A is found in CNS barrier endothelium [66]. Here, MFSD2A is not merely an endothelial transporter or marker; it contributes to the low-vesicular-traffic program that distinguishes the mature BBB [67, 68]. Genetic studies identify Mfsd2a as essential for BBB formation and function, with barrier leakage arising from excess vesicular transport across brain endothelium [6, 69]. Vesicular transcytosis provides a direct and reproducible link between transporter activity and barrier physiology.

BBB maturation coordinates junctional organization, transporter expression, lipid composition, and suppression of vesicular traffic [6]. MFSD2A is integrated into this broader specialization program. SOX18-driven maturation of induced brain microvascular endothelial cells increases Mfsd2a together with endothelial, tight-junction, and BBB-transporter markers, whereas endothelial-pericyte co-culture raises MFSD2A protein relative to monoculture [44, 45, 70]. These model systems indicate that developmental and cellular cues regulate MFSD2A expression, but do not identify a single upstream factor as sufficient to determine its in vivo state [71].

Across diverse permeability models, lower MFSD2A is repeatedly associated with greater transcellular transport [71]. This pattern includes increased vesicular or caveolae-mediated transcytosis, enhanced CAV1/caveolin-1 features, and higher permeability [71]. Chronic cerebral hypoperfusion, subarachnoid hemorrhage, endothelial PTEN loss, traumatic brain injury, hypertension-related vascular stress, gingipain exposure, endotoxemia, and angiotensin II infusion each link altered Mfsd2a/MFSD2A to one or more barrier abnormalities [7, 8, 72–79]. Their initiating mechanisms differ, but the recurrence of the transcytosis phenotype underscores the physiological importance of the MFSD2A-associated endothelial state.

Transcytosis is a particularly informative endpoint because it distinguishes MFSD2A biology from nonspecific endothelial injury [71]. Many disease models simultaneously perturb tight junctions, inflammatory signaling, metabolism, and vascular support cells [80, 81]. By contrast, MFSD2A-associated control of caveolae and vesicular traffic defines a more specific route through which membrane lipid state can alter transcellular permeability [7, 69, 73, 75]. This mechanistic focus does not diminish paracellular failure; it clarifies that complex barrier injury may combine transcellular, junctional, inflammatory, and hemodynamic mechanisms.

Restoration experiments provide directionality, although not therapeutic validation [71]. Mfsd2a overexpression reduces vesicular transcytosis during chronic cerebral hypoperfusion and reverses CAV1-associated changes after subarachnoid hemorrhage [7, 73]. Other interventions that increase MFSD2A or modify the MFSD2A/Cav-1 axis have been accompanied by less BBB leakage, lower Cav-1, or improved barrier readouts [81–85]. These findings support a modifiable relationship in defined models. They do not establish pharmacological activation as a clinical strategy, nor do they show that deliberate manipulation would spare the healthy BBB, blood-tumor barrier, retina, placenta, or other endothelial beds.

Regulation of MFSD2A is likewise context dependent [86]. Endothelial maturation, pericyte contact, Wnt/β-catenin-linked signaling during inflammatory injury, vascular stress, and experimental interventions that alter Cav-1 or permeability have each been associated with the MFSD2A barrier state [44, 45, 78, 79]. These observations are better viewed as inputs into a broader endothelial program than as components of a single linear pathway. The available evidence places MFSD2A within networks that coordinate transcytosis and barrier maturation [71].

This endothelial-state model explains why MFSD2A appears in both developmental and adult-injury studies. During BBB formation, MFSD2A is required for acquisition of the low-transcytosis phenotype [6, 69]. In mature or injured endothelium, reduced expression may mark loss of specialization, contribute to increased permeability, or both [7, 73, 87]. Developmental requirement and injury-associated dysregulation are related but not equivalent: the former establishes necessity for normal barrier formation, whereas the latter describes a component of barrier breakdown [88].

Distinguishing marker, mediator, and modulator is particularly important in disease models [89]. MFSD2A acts as a marker when its expression tracks with acquisition or loss of a BBB-like endothelial phenotype [6]. It functions as a mediator when gain or loss changes LPC uptake, endothelial lipid composition, caveolae, and permeability [7, 8, 75]. It is best described as a modulator when an intervention changes MFSD2A alongside other barrier components. This vocabulary makes clear why some studies support a causal mechanism, whereas others establish association or experimental context [90].

**Fig. 2:**
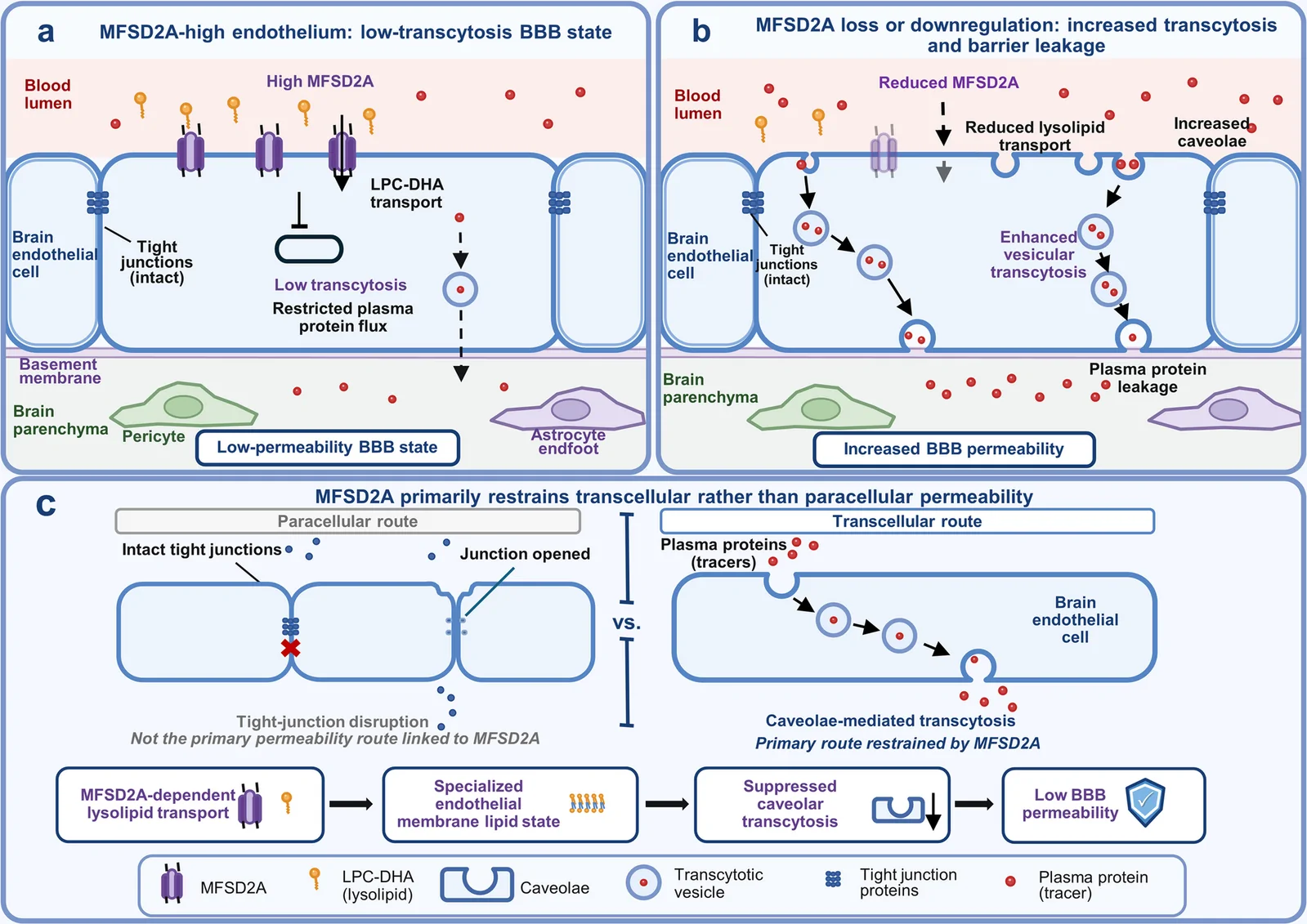
MFSD2A-dependent suppression of Blood–Brain Barrier transcytosis. **a** Brain endothelial cells with high MFSD2A activity. MFSD2A-dependent LPC-DHA transport supports a specialized membrane lipid state characterized by low vesicular transcytosis, restricted plasma-protein flux, and low Blood–Brain Barrier permeability; tight junctions remain intact. **b** MFSD2A loss or downregulation. Reduced lysolipid transport is associated with increased caveolar or vesicular transcytosis, greater transendothelial passage of plasma proteins, and increased permeability, even when interendothelial junctions remain morphologically closed. **c** Transcellular versus paracellular permeability. MFSD2A most directly restrains caveolae-mediated transcellular transport rather than tight-junction opening. The proposed mechanism links MFSD2A-dependent lipid transport to an endothelial membrane environment that suppresses caveolae and maintains low BBB permeability. Disease states may nevertheless involve concurrent paracellular defects. BBB, Blood–Brain Barrier; LPC-DHA, lysophosphatidylcholine carrying docosahexaenoic acid

A parsimonious barrier model comprises two linked layers [91]. First, MFSD2A-mediated LPC uptake shapes the membrane lipid environment of brain endothelial cells [2]. Second, this lipid state restrains caveolae formation and vesicular transcytosis [69, 92]. Developmental and injury studies can be interpreted against this sequence: reduced MFSD2A may alter lipid composition, release vesicular restraint, or do both [93]. Conversely, restoration or overexpression is associated with lower permeability in selected models [7, 8, 94, 95]. Figure 2 distinguishes MFSD2A-associated transcellular restraint from paracellular junctional failure.

### MFSD2A-dependent lipid transport and endothelial lipid homeostasis

The low-transcytosis phenotype of CNS endothelium is rooted in MFSD2A-dependent lipid transport [96]. At the BBB, endothelial MFSD2A imports DHA and other fatty acids in LPC-bound form [2, 23, 97]. This places lipid acquisition, rather than MFSD2A expression alone, at the center of barrier physiology. By coupling circulating lysolipids to endothelial membrane composition, the transporter provides a mechanistic route from substrate uptake to caveolar restraint and barrier integrity [96].

Loss-of-function studies make this requirement explicit [98]. Mfsd2a-deficient mice show markedly reduced uptake of LPC-DHA and other LPC species into the brain, accompanied by lower brain DHA [2, 99]. Elevated circulating LPC species in knockout animals and affected individuals are consistent with impaired tissue uptake, and patient lipid profiles similarly indicate reduced utilization of mono- and polyunsaturated LPC substrates [9]. Endothelial-specific deletion further localizes this requirement to the postnatal BBB: deficient DHA accretion precedes impaired brain growth and microcephaly in the tested model [10].

Endothelial lipid homeostasis offers a cellular explanation for the control of transcytosis [100, 101]. Uptake of LPC-bound unsaturated fatty acids through MFSD2A alters the membrane lipid environment in a manner associated with fewer caveolae and preserved BBB integrity [8, 75, 102]. In experimental subarachnoid hemorrhage, Mfsd2a overexpression has been linked to DHA delivery, caveolar suppression, and reduced vesicular trafficking [7]. These data support a lipid-dependent route from transport to barrier phenotype, while leaving room for parallel junctional, inflammatory, vascular, and injury-specific mechanisms.

MFSD2A-dependent transport also intersects with broader lipid-metabolic programs, although the causal depth of these links varies. Reduced DHA delivery has been associated with altered SREBP-1-dependent lipogenesis in Mfsd2a-knockout models, whereas injury studies have connected Mfsd2a overexpression and DHA with SREBP1-related endothelial changes and vesicular traffic [8, 103]. These observations show that substrate transport can reshape lipid metabolism. They do not establish direct control of ACSL4/LPCAT3, lipid peroxidation, or ferroptosis, and should not be used as shorthand for a general lipid-death pathway.

The most defensible mechanistic sequence is LPC or LPC-DHA transport, altered substrate availability, a change in endothelial membrane state, suppression of caveolae, and stabilization of tissue-level barrier function [2, 7, 23, 75]. Support for this sequence is strongest at CNS barriers and less complete elsewhere. Future studies should connect transporter activity to defined lipid species, membrane biophysical properties, vesicular traffic, and tissue outcomes within the same experimental system. Until then, downstream pathway labels should remain subordinate to the established transport-barrier axis.

The same discipline applies to dietary and substrate-supply claims [104, 105]. MFSD2A transports LPC-bound fatty acids, and lipid availability is critical in several tissues, but substrate delivery is not itself a validated intervention [23, 101, 106, 107]. Brain, retina, placenta, and developing organs differ in access route, developmental timing, transporter abundance, and compensatory metabolism. Dietary or carrier-based findings are informative only when the relevant tissue, MFSD2A state, and lipid endpoint are specified [105, 108–110]. They do not support a general recommendation to increase PUFA intake or pharmacologically activate MFSD2A in disease. Studies of phosphatidylcholine-DHA and dietary LPC-EPA support the importance of carrier form for brain lipid delivery, but do not establish disease-specific MFSD2A therapy [111, 112].

### Tissue-specific lipid transport contexts beyond the BBB

MFSD2A physiology extends beyond the BBB [61, 63]. The retina, placenta, alveolar epithelium, kidney, epidermis, and immune cells each use MFSD2A-associated lipid transport in settings where local function depends on substrate availability or membrane composition [1, 23, 35, 113]. Together, these tissues support broad deployment of a lysolipid transporter. They do not support the assumption that every site shares the BBB phenotype, the same disease vulnerability, or a common therapeutic window.

The retina provides the closest barrier-adjacent example and is tightly linked to DHA biology [114, 115]. Genetic and transport studies implicate MFSD2A in retinal LPC-DHA acquisition, photoreceptor support, and maintenance of retinal lipid composition [1, 23, 116, 117]. The eye and brain share a dependence on barrier-associated lipid delivery, but differ in anatomy, cellular organization, and disease context. Retinal evidence strengthens the transport-homeostasis model without establishing the safety of BBB drug delivery or a clinical nutritional intervention.

The placenta introduces a distinct maternal–fetal transport interface [61, 63, 118]. MFSD2A expression has been associated with placental DHA transfer and cord-blood DHA, while studies in gestational diabetes report lower placental or maternal MFSD2A alongside less efficient transfer [108, 109, 113, 119, 120]. These findings place MFSD2A within developmental lipid supply outside neural barriers. They do not validate dietary therapy or pharmacological activation, nor do they imply that maternal–fetal transport predicts the response of CNS endothelium to manipulation.

Lung, kidney, epidermis, and immune cells illustrate still greater functional divergence [121, 122]. In alveolar type II cells, MFSD2A-dependent LPC uptake contributes to surfactant lipid composition [35, 36]. In S3 proximal-tubule cells, localization, in vivo probe activity, and altered DHA-containing phospholipids in haploinsufficient animals support a renal transport role [37]. Differentiated keratinocytes use MFSD2A to incorporate circulating LPC into epidermal phospholipid pools. Activated T cells likewise depend on MFSD2A for LPC uptake, but this finding alone does not establish immunotherapy response or reversal of T-cell exhaustion [3].

**Fig. 3:**
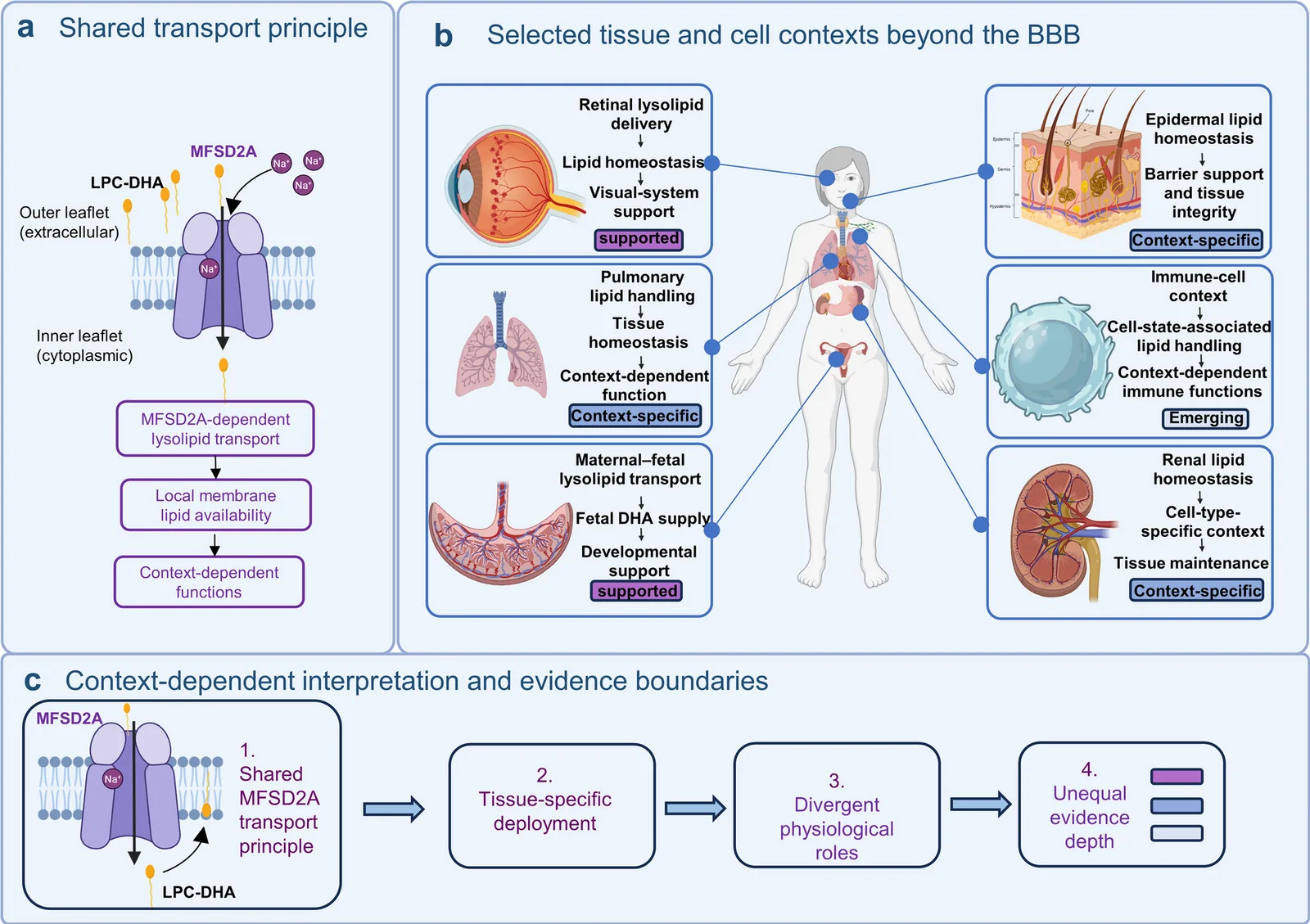
Tissue-specific MFSD2A-mediated lysolipid transport beyond the blood–brain barrier. **a** Shared transport principle. Across tissues, MFSD2A mediates Na⁺-dependent transfer of LPC-DHA from the extracellular-facing to the cytoplasmic-facing membrane leaflet, thereby altering local lysolipid availability. **b** Selected tissue and cellular contexts. Relatively well-supported functions include retinal lysolipid delivery and visual-system lipid homeostasis, as well as maternal–fetal transfer that contributes to fetal DHA supply. Studies in lung, skin, kidney, and selected immune-cell populations indicate additional roles in tissue-specific lipid handling, although the type and depth of evidence vary. These functions should not be interpreted as replicas of the brain endothelial mechanism. **c** Interpretive framework beyond the BBB. A shared molecular transport process is deployed in distinct cellular environments and produces divergent physiological outputs. Mechanistic and translational conclusions must be calibrated to tissue context, experimental system, and evidence strength. BBB, blood–brain barrier; LPC-DHA, lysophosphatidylcholine carrying docosahexaenoic acid

Across these physiological systems, the shared mechanism remains narrowly defined as tissue-specific handling of LPC-bound fatty acids [1, 23, 35, 37, 113]. They do not support a universal disease pathway. Table 1 compares the relevant cell types, substrates and lipid readouts, experimental approaches, physiological functions, and permissible strength of interpretation. Figure 3 summarizes the shared transport principle and tissue-specific evidence boundaries.

**Table 1:** Tissue- and cell-context evidence for MFSD2A-mediated lysolipid transport and physiological function

Tissue or barrier context	MFSD2A-expressing cell type and localization	Principal evidence and readouts	Supported transport or lipid-handling function	Established physiological role	Phenotype after MFSD2A disruption	Evidence tier and permitted inference	Key references
Central nervous system vascular barrier/blood–brain barrier (BBB)	Brain microvascular endothelial cells; MFSD2A is expressed at the plasma membrane as part of the specialized BBB endothelial program	Global and endothelial-specific loss-of-function models; LPC and LPC-DHA uptake assays; plasma and brain lipid measurements; electron-microscopic vesicle counts; tracer-permeability studies	Na⁺-dependent uptake and flipping of LPC-bound fatty acids, especially LPC-DHA; establishment of an endothelial lipid state that restrains caveolae-mediated transport	Brain DHA accretion, postnatal brain growth, BBB maturation, and maintenance of low transcellular permeability	Reduced brain LPC-DHA uptake and DHA content; elevated circulating LPC species; increased caveolar transcytosis and BBB leakage; microcephaly in defined developmental models	E1, established. Direct transport-to-barrier and transport-to-development relationships are supported. MFSD2A is neither the sole cause of BBB failure nor primarily a tight-junction regulator	[10, 79, 82, 123]
Retina and blood-retinal barrier (BRB)	Retinal vascular endothelium and the retinal LPC-DHA delivery pathway that supports photoreceptor lipid supply	Mfsd2a loss or transport-disruption studies; retinal lipid and DHA measurements; photoreceptor phenotyping; developmental and injury-related analyses of BRB permeability and transcytosis	Delivery of LPC-bound DHA to the retina, maintenance of retinal membrane lipids, and restriction of endothelial transcytosis at the BRB	Retinal DHA supply, photoreceptor development and maintenance, and acquisition of a low-transcytosis BRB state	Reduced retinal LPC-DHA delivery, altered retinal lipids, impaired photoreceptor integrity, and increased BRB permeability in selected developmental or injury models	E1 for retinal lysolipid transport; E2 for disease-associated BRB modulation. Ocular data do not establish healthy-BBB delivery safety or a general nutritional intervention	[1, 46, 72, 98]
Placenta/maternal–fetal interface	Placental trophoblast and syncytiotrophoblast compartments. MFSD2A also serves as the receptor for syncytin-2, a function distinct from lysolipid transport	Placental expression studies; maternal, placental, and cord-blood DHA measurements; gestational-diabetes and fetal-growth-restriction cohorts; placental knockdown in pregnant mice; trophoblast-fusion assays	MFSD2A-associated handling of LPC-DHA and contribution to maternal–fetal DHA transfer; a separate receptor function in trophoblast fusion	Fetal lipid supply, fetal-brain phospholipid DHA accretion, and placental trophoblast development	Lower cord-blood or fetal DHA in selected pregnancy contexts; reduced fetal brain growth and phospholipid DHA after placental knockdown. Associations vary with maternal and placental condition	E2, context-supported. The evidence supports placental lipid transfer and development, but not dietary treatment, pharmacological activation, or direct equivalence to BBB biology	[91, 93, 99, 100]
Lung/alveolar surface	Alveolar type II epithelial cells; MFSD2A is localized predominantly to the apical membrane facing the alveolar lining fluid	Cell-surface localization; LPC-uptake assays; genetic perturbation; surfactant-lipid profiling; pulmonary phenotyping	Uptake and recycling of LPC generated at the alveolar surface, supporting surfactant phospholipid composition	Pulmonary surfactant lipid homeostasis and alveolar epithelial function	Altered surfactant lipid composition and pulmonary homeostasis after MFSD2A disruption; injury-related findings remain model specific	E2, context-supported. A tissue-specific lipid-handling function is supported; pulmonary disease mechanisms should not be generalized beyond the tested models	[35, 36]
Kidney/proximal tubule	S3 proximal-tubule epithelial cells; MFSD2A is reported at the basolateral membrane	In vivo lysolipid-probe transport; Mfsd2a haploinsufficiency or perturbation; renal lipidomics; recovery after acute kidney injury	Local LPC-DHA transport and maintenance of DHA-containing renal phospholipid pools	Proximal-tubule lipid homeostasis and recovery after acute kidney injury	Altered DHA-containing phospholipids and impaired or delayed renal recovery in defined models	E2, context-supported. The evidence supports a renal transport-and-repair function, not a universal kidney-disease biomarker or an established therapy	[37, 102]
Epidermis/skin	Differentiated epidermal keratinocytes; MFSD2A is expressed at the plasma membrane in the mature epidermal compartment	Cell-type-specific genetic disruption; uptake of plasma-derived LPC; epidermal phospholipid profiling; tissue-homeostasis phenotyping	Import of circulating LPC species into epidermal phospholipid pools	Epidermal lipid composition, differentiation, and tissue homeostasis	Reduced LPC uptake, altered epidermal phospholipid pools, and loss of normal epidermal homeostasis	E2, context-supported. The findings establish a differentiated epithelial lipid-uptake pathway but do not reproduce the BBB transcytosis mechanism in skin	[23]
Activated and memory CD8⁺ T cells	Activated and memory CD8⁺ T cells; MFSD2A-dependent uptake occurs at the immune-cell plasma membrane	Genetic loss-of-function; fluorescent LPC-uptake assays; memory-cell maintenance; secondary-response experiments	LPC uptake that supports lipid supply during T-cell activation and memory maintenance	Maintenance of CD8⁺ memory T cells and effective secondary responses to infection	Reduced LPC uptake and impaired maintenance or secondary function of CD8⁺ memory T cells	E2, context-supported. The evidence establishes immune-cell transport biology, not pan-cancer immunotherapy prediction, reversal of T-cell exhaustion, or a universal tumor-immune mechanism	[3]

This table compares localization, experimental readouts, supported functions, phenotypes, and permitted evidence strength across tissues

Evidence tiers are defined here as follows: E1, established; E2, context-supported; E3, exploratory or associative; and boundary-only, insufficient for a positive mechanistic or translational claim. These categories are interpretive rather than formal clinical grades. *BBB* blood–brain barrier, *BRB* blood-retinal barrier, *DHA* docosahexaenoic acid, *LPC* lysophosphatidylcholine

Such comparisons also expose the limitations of the term ‘barrier’. CNS endothelium is defined by low transcytosis; the blood-retinal barrier contains distinct ocular compartments; the placenta mediates maternal–fetal exchange; the alveolar surface maintains surfactant; and the epidermis and kidney are epithelial systems with their own lipid-uptake requirements [6, 35, 37, 113]. MFSD2A contributes to lipid handling in each setting, but perturbation will not produce an identical phenotype. Tissues are best compared by cellular localization and transport context rather than by barrier nomenclature alone [124, 125].

Developmental and nutritional interpretations require the same precision [116, 126]. Placental studies support a role for MFSD2A in fetal DHA transfer, whereas retinal and brain studies establish the importance of LPC-DHA uptake in tissues with high lipid demand [23, 108, 109, 113]. These observations do not combine into a simple nutritional rescue model. Substrate supply, transporter capacity, developmental timing, tissue demand, and disease state all influence whether additional lipid can be used [34, 118]. Table 1 emphasizes the measured transport or lipid endpoint and the level of inference that each tissue context can sustain.

## MFSD2A in inherited and acquired disease

MFSD2A enters disease biology most convincingly through neurodevelopment [127]. Human biallelic variants and experimental Mfsd2a loss connect defective lysolipid transport to microcephaly-spectrum phenotypes, impaired DHA accretion, abnormal brain growth, and barrier dysfunction [10, 128]. A second, mechanistically distinct layer comes from acquired vascular injury, in which the MFSD2A-associated endothelial state is destabilized by ischemic, inflammatory, or other stressors [7, 73, 129]. Together, these settings establish disease relevance without making MFSD2A a universal neurological disease gene or an already validated therapeutic target.

### Human genetics and microcephaly syndromes

Human genetics provides the clearest clinical evidence for MFSD2A-related disease [130]. Biallelic pathogenic variants have been identified in rare primary microcephaly syndromes, including autosomal-recessive primary microcephaly 15 and non-lethal microcephaly phenotypes [128, 131–133]. These reports support a recessive disorder in which loss of lysolipid-transporter function compromises brain growth and neurodevelopment. Their rarity and specificity are equally important: MFSD2A is a defined disease locus, not a general explanation for neurological disease or a population-level biomarker.

The reported variants arise in more than one family and ascertainment setting [130]. A homozygous missense allele was identified in siblings from a shared-ancestry family, targeted-exome sequencing implicated MFSD2A in microcephaly, and additional unrelated individuals carried novel homozygous pathogenic variants with broader clinical features [128, 131, 132]. This recurrence supports pathogenicity beyond a single pedigree, but natural history, penetrance, age-dependent progression, genotype–phenotype relationships, and biomarker utility remain poorly defined. Systematic comparison of variant class, residual transport, lipid phenotype, and clinical severity will be needed to resolve these questions [134].

The phenotype extends beyond reduced head circumference [130]. Reported manifestations include non-lethal disease, global neurodevelopmental abnormalities, and microcephaly with hypomyelination [131, 132]. The white-matter phenotype is consistent with disrupted lipid supply during brain development and raises the possibility of glial as well as vascular contributions [103]. Biological coherence, however, does not identify a single downstream cell type or mechanism. The patient data establish a syndromic association and motivate mechanistic studies; they do not resolve the full cellular basis of disease [135].

Plasma lipid measurements provide a biochemical bridge between genotype and transport [136]. Affected individuals show increased LPC species carrying mono- and polyunsaturated fatty acids, a pattern interpreted as reduced uptake of MFSD2A substrates into the brain [9]. This finding links the syndrome to defective lysolipid handling rather than to an undifferentiated neurodevelopmental phenotype. It has not been validated as a diagnostic biomarker and does not establish ACSL4/LPCAT3 coupling, ferroptosis regulation, or a lipid-based therapy [103].

Important variant-level mechanisms remain unresolved [137]. Individual alleles may alter folding, membrane localization, sodium coupling, substrate recognition, or transport kinetics, but these effects have not been mapped systematically in patient-relevant cells. It is also unclear whether clinical severity is driven primarily by impaired endothelial substrate entry, reduced neuronal or glial lipid availability, defective myelination, altered barrier maturation, or a combination of these processes. These uncertainties define the next stage of genotype–phenotype analysis rather than weakening the established link to rare neurodevelopmental disease [103, 128, 131, 132].

Comparative genetics can support, but not replace, the human evidence. A frameshift variant in MFSD2A was proposed as the cause of microcephaly in Kerry Hill sheep because the phenotype resembled human and mouse loss-of-function states and the allele was absent from more than 1,000 control genomes [138]. Such models can help dissect transport, endothelial contribution, and developmental timing, but should be treated as comparative evidence rather than independent proof of clinical generalizability.

### Experimental neurodevelopmental phenotypes

Experimental models provide causal resolution that rare human pedigrees cannot supply on their own [139]. By varying cell type and developmental timing, they can separate transporter loss from secondary changes in substrate availability, barrier maturation, and tissue growth. The most informative studies couple Mfsd2a disruption to measured DHA accretion, neurodevelopmental outcomes, and barrier phenotypes [10, 131]. Together, they support a transport-development model in which impaired lysolipid entry at the vascular interface limits brain lipid supply during a period of rapid growth.

Endothelial deletion experiments provide the clearest temporal and cellular evidence [140]. Constitutive and inducible vascular endothelial deletion of Mfsd2a demonstrates a postnatal requirement at the BBB for normal DHA accretion and brain growth [141]. Restricting the perturbation to endothelium localizes a causal step to the vascular interface, whereas inducible deletion separates this postnatal requirement from earlier embryonic effects [142]. In this model, DHA deficiency precedes microcephaly, supporting the sequence from impaired endothelial transport to deficient lipid accretion and reduced brain growth [10].

Zebrafish and whole-animal loss-of-function models extend this developmental relationship across species [143]. Morpholino knockdown of an MFSD2A orthologue impairs LPC uptake and causes severe microcephaly with disruption of the BBB [131]. In another model, Mfsd2a ablation produces postnatal microcephaly, motor dysfunction, increased barrier permeability, growth failure, and poor survival [123, 144]. These phenotypes reinforce the transport-development axis, but their severity and species-specific features limit direct extrapolation to the human syndrome.

Biochemical flippase studies provide the molecular mechanism beneath these developmental phenotypes [145]. Mfsd2a mediates sodium-dependent movement of lysolipids from the outer to the inner membrane leaflet, as supported by fluorescence-based and ionophore experiments [22, 146]. This evidence establishes MFSD2A as an active transporter rather than a gene merely associated with microcephaly. Claims about brain growth, myelination, survival, motor function, or barrier maturation nevertheless require phenotype-specific evidence in the relevant model [147].

Experimental studies therefore sharpen rather than broaden the disease claim [148]. In defined systems, MFSD2A loss causes impaired substrate uptake or DHA accretion and is followed by abnormal brain growth, neurodevelopment, or barrier maturation [10, 123, 131]. These elements constitute the established causal core [149]. The contributions of individual neural and glial cell types, compensatory lipid pathways, long-term clinical progression, and the feasibility of correcting a developmental substrate deficit remain unresolved [150].

Timing is likely to be decisive [151]. Brain growth, BBB maturation, myelination, retinal development, and systemic lipid metabolism follow different developmental trajectories [152]. Loss of transport during a critical window may produce effects that cannot be reproduced by adult inhibition or reversed by late substrate supplementation [10, 23, 103]. Future rescue studies must distinguish developmental requirement from maintenance function and targeted correction from nonspecific lipid loading.

**Fig. 4:**
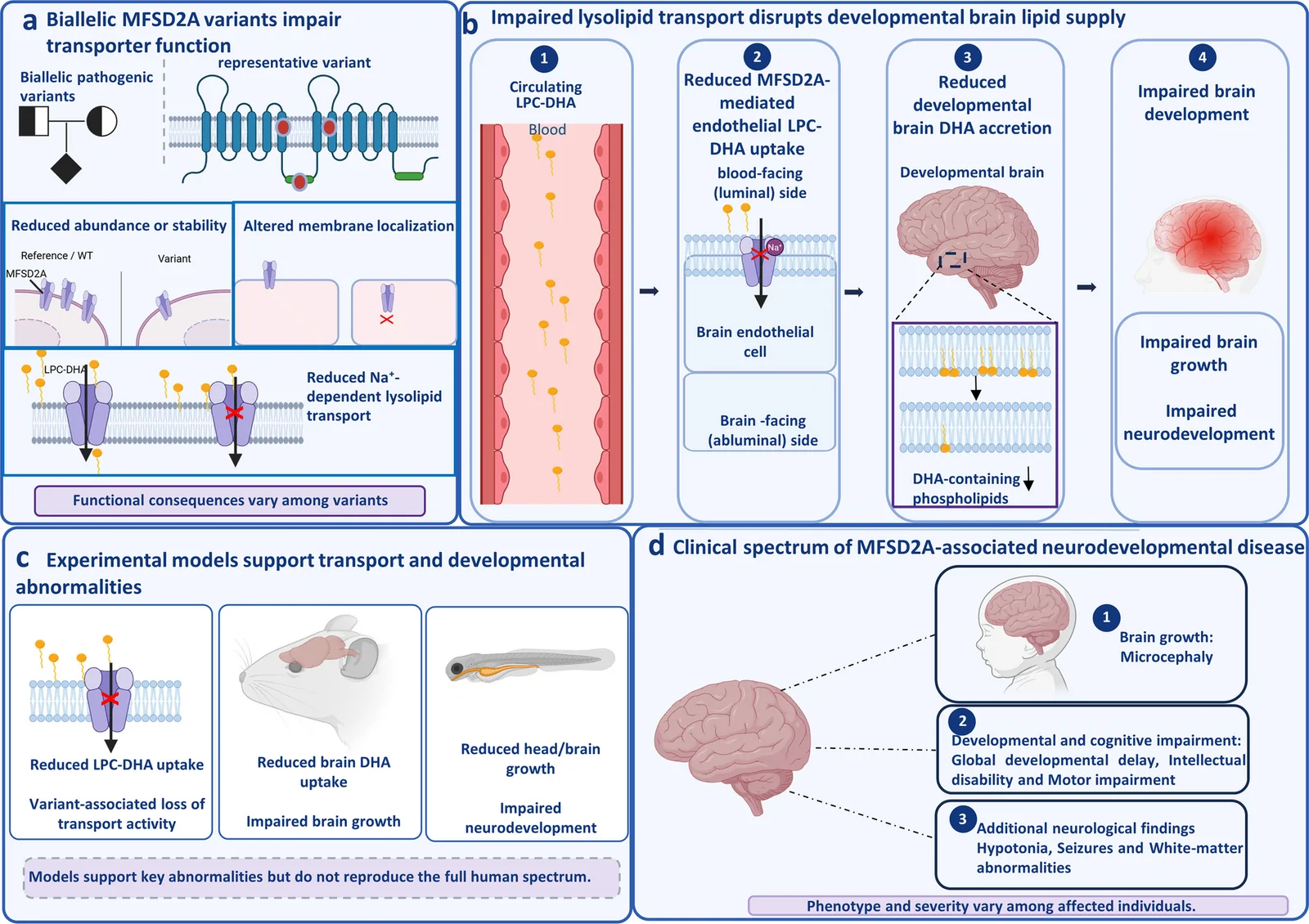
MFSD2A variants, impaired lysolipid uptake and neurodevelopmental disease. **a** Biallelic pathogenic MFSD2A variants. Variant-dependent defects may involve protein abundance or stability, membrane localization, and Na⁺-dependent lysolipid transport. The positions shown are representative and do not imply a uniform molecular effect or a quantitative relationship between variant location and clinical severity. **b** Proposed pathogenic sequence. Reduced endothelial uptake of circulating LPC-DHA limits developmental brain DHA accretion and incorporation into DHA-containing phospholipids, thereby restricting membrane lipid supply required for normal growth and neurodevelopment. **c** Experimental support. Cellular assays and complementary animal models reproduce individual steps, including reduced LPC-DHA uptake, lower brain DHA, impaired brain growth, and neurodevelopmental abnormalities, but no single model captures the full human syndrome. **d** Clinical spectrum. Microcephaly is the predominant feature and may be accompanied by global developmental delay, intellectual disability, motor impairment, hypotonia, seizures, and white-matter abnormalities. Phenotypic severity varies among affected individuals, consistent with genetic heterogeneity and differences in residual transporter function. LPC-DHA, lysophosphatidylcholine carrying docosahexaenoic acid; WT, wild type

No single experimental system captures the full disorder [153]. Endothelial deletion localizes the transport requirement to the vascular interface [10]. Zebrafish knockdown and whole-animal ablation reveal organism-level consequences [123, 131]. Biochemical assays define sodium-dependent lysolipid transport [22]. Patient studies establish the human microcephaly-spectrum association [9]. Their convergence supports a coherent sequence from transporter dysfunction to limited lipid availability and neurodevelopmental impairment, while leaving the clinically dominant step uncertain for each allele and developmental stage. Figure 4 links variant-level transport defects to developmental phenotypes while preserving genotype–phenotype heterogeneity.

This convergence is precisely why MFSD2A cannot be understood through rare genetics or endothelial biology alone. Patient studies establish clinical relevance, developmental models supply causality, biochemical assays define protein function, and BBB studies identify a privileged route for lipid entry into the brain [9, 10, 22, 128]. Integrating these layers reveals where evidence is mutually reinforcing and where mechanistic gaps remain, without implying that every phenotype has been fully explained.

### Acquired barrier dysfunction: linking physiology to disease

Acquired barrier injury poses a different problem from inherited transporter deficiency: how an MFSD2A-dependent endothelial state responds to vascular, inflammatory, or parenchymal stress [71]. Across several models, reduced or dysregulated Mfsd2a/MFSD2A accompanies increased BBB permeability and loss of the low-vesicular-traffic phenotype [7, 73, 75, 154]. These findings connect core BBB physiology to disease, but the initiating insults remain model specific and cannot be reduced to MFSD2A loss alone [76, 155].

The strongest acquired-disease examples link MFSD2A directly to transcellular permeability [71]. Chronic cerebral hypoperfusion reduces hippocampal Mfsd2a and increases vesicular transcytosis and BBB leakage [73]. After subarachnoid hemorrhage, reduced Mfsd2a is associated with barrier disruption, whereas upregulation limits endothelial transcytosis and improves permeability readouts [7]. Endothelial PTEN loss provides a complementary genetic context in which reduced MFSD2A increases caveolae-mediated transcellular transport [75].

Caveolae and vesicular trafficking provide the mechanistic link to canonical BBB biology [71]. Mfsd2a-deficient mice have more caveolar vesicles in CNS endothelium and increased permeability, establishing a reference phenotype for acquired injury [69]. Chronic hypoperfusion, hypoxic-ischemic injury, traumatic brain injury, gingipain exposure, endotoxemia, angiotensin II, and other vascular stressors extend this pattern by associating Mfsd2a status with vesicular transport, Cav-1, junctional stress, or permeability [73, 77–79, 156]. What these conditions share is disruption of an endothelial transport state, not a common disease pathway. Broader BBB studies of ischemia, hyperglycemia, heart failure, glycocalyx loss, pericyte coverage, and transcellular routes provide context for these phenotypes without making MFSD2A their universal initiating cause [48, 51, 53–57, 157–159].

Upstream signals should be interpreted separately. Wnt/β-catenin activity, angiotensin-related inflammation, pericyte and mural-cell support, and injury-associated changes in Cav-1 each intersect with the MFSD2A barrier state in particular models [45, 74, 78, 79]. They are neither equivalent nor components of a single established cascade. Their relevance is best judged by the experimental perturbation, the cell type involved, and whether MFSD2A transport, expression, or only a downstream permeability readout was measured [83, 84, 160].

Inherited deficiency and acquired barrier injury thus converge on the same transporter-barrier module from opposite causal directions. In genetic disease, MFSD2A dysfunction is upstream of deficient lipid delivery and abnormal development [10]. During vascular or inflammatory injury, MFSD2A may instead be downregulated or functionally impaired as part of a broader endothelial stress response [7, 73, 75, 161]. Recognizing this difference prevents mechanistic continuity from being mistaken for causal equivalence.

**Fig. 5:**
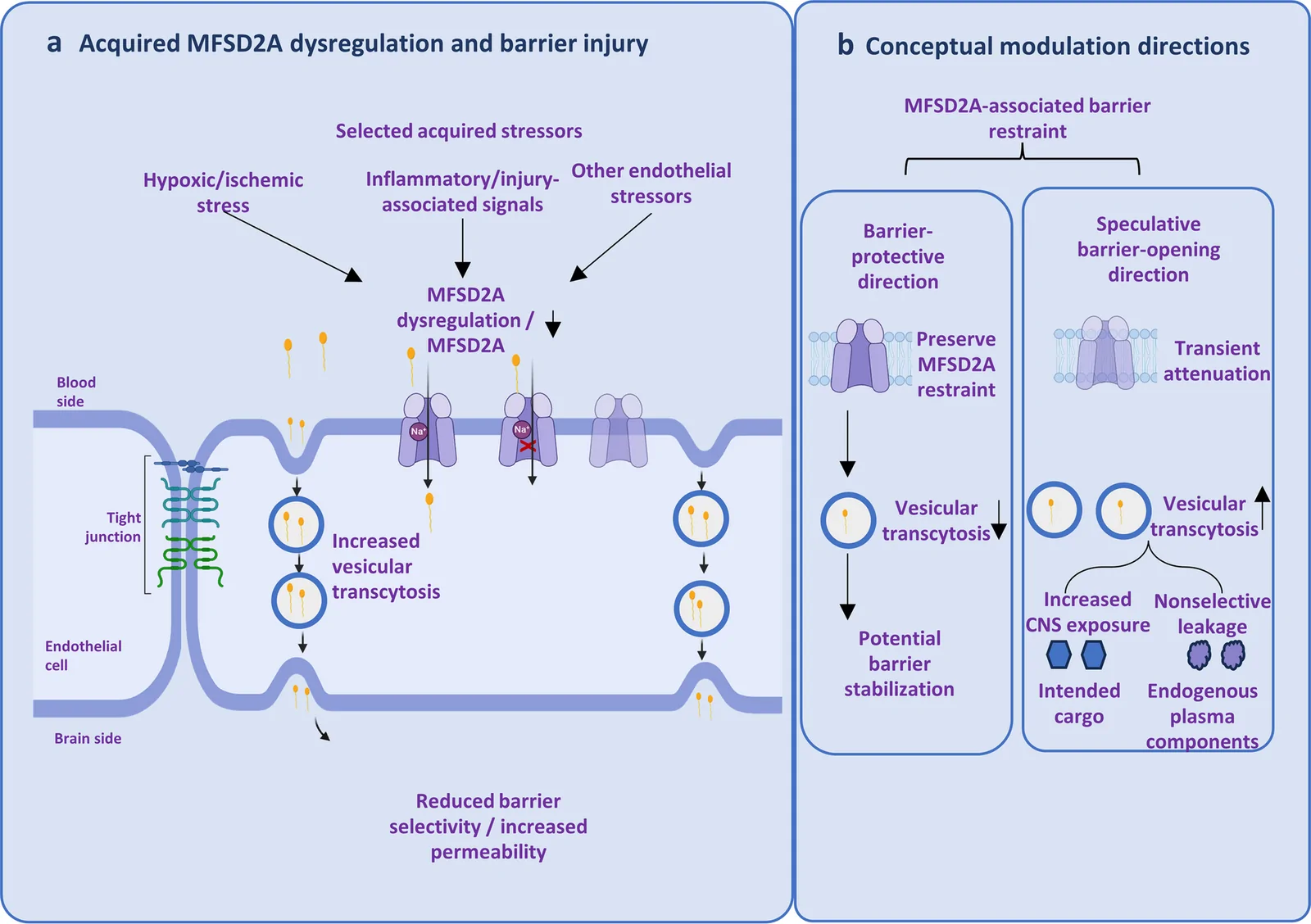
Acquired MFSD2A dysregulation and barriers to therapeutic modulation. **a** Acquired endothelial dysregulation in selected barrier-injury settings. Hypoxic or ischemic stress, inflammatory or injury-associated signals, and other endothelial stressors may reduce MFSD2A abundance or activity. Loss of the MFSD2A-associated membrane state is linked to increased vesicular transcytosis, lower barrier selectivity, and greater permeability, while interendothelial tight junctions remain closed in the pathway illustrated. This relationship has been observed in specific models and is not presented as a universal mechanism of BBB injury. **b** Opposing concepts for therapeutic modulation. Preserving MFSD2A-associated vesicular restraint could support barrier stabilization, whereas transient attenuation has been proposed to increase CNS exposure to a selected cargo. The latter may also permit nonselective entry of endogenous plasma constituents. Neither approach is clinically validated, and the required specificity, timing, magnitude, reversibility, and safety remain unresolved. CNS, central nervous system

The connection is biologically informative but not yet translationally mature [162]. Experimental data support a role for MFSD2A in barrier states characterized by altered caveolae, vesicular traffic, and permeability [7, 69, 73, 75]. They do not establish MFSD2A loss as the universal origin of BBB failure, nor do they validate activation, intentional barrier opening, drug delivery, dietary LPC-DHA, or selective manipulation of the blood-tumor barrier. These possibilities require direct intervention, reversibility, and safety studies [163]. Figure 5 separates barrier-preserving and barrier-opening concepts and their distinct safety requirements.

### Chronic neurodegenerative disorders

Links between MFSD2A and chronic neurodegeneration remain biologically plausible but causally weak. Retinal and vascular studies in Alzheimer-type models report altered MFSD2A expression, AQP4, DHA-related readouts, or barrier phenotypes, and a small human study reported lower circulating MFSD2A in Alzheimer’s disease [49, 50, 115, 149, 150, 164]. These observations arise from different tissues, assays, and disease stages and cannot be combined into a single disease mechanism. Reduced DHA-containing phospholipids or BBB dysfunction may accompany neurodegeneration without proving that MFSD2A initiated the process.

A recent experimental amyotrophic lateral sclerosis study reported benefit from combined Mfsd2a overexpression and DHA supplementation in a defined model [165]. This finding is hypothesis-generating and disease specific; it does not establish a general nutritional rescue strategy, clinical efficacy, or a shared mechanism across neurodegenerative disorders. Current evidence does not establish MFSD2A as a causal driver, diagnostic biomarker, or validated therapeutic target in Alzheimer’s disease or other chronic neurodegenerative disorders.

### Ocular disease

Ocular evidence now extends beyond physiological retinal DHA delivery. MFSD2A supports lysolipid transport and a low-transcytosis state at the blood–retinal barrier, whereas the 2026 glaucoma study linked elevated intraocular pressure to reduced endothelial MFSD2A, increased transcytosis, and focal barrier leakage that preceded neurodegeneration in mouse models. The study also detected ganglion-cell-layer leakage in 14 of 17 human primary open-angle glaucoma eyes, while endothelial β-catenin stabilization reduced leakage and neuronal loss in the experimental system [1, 68, 92, 114–117]. These findings establish disease-associated barrier instability, not a clinically validated MFSD2A-directed intervention.

### Inflammatory, metabolic, and organ-injury contexts

MFSD2A can participate in inflammation resolution when the relevant cell type, substrate, and lipid products are demonstrated. In intestinal microvascular endothelium, MFSD2A-dependent DHA handling supported the generation of pro-resolving lipid mediators, and MFSD2A-overexpressing endothelial progenitor cells reduced colitis in mice [18]. This is mechanistic and preclinical evidence; it does not establish efficacy in human inflammatory disease.

Organ injury illustrates why transporter activity is not intrinsically protective or harmful. Renal MFSD2A-dependent lysolipid transport supports restoration of DHA-containing phospholipids and recovery after acute kidney injury, whereas pulmonary MFSD2A contributes to surfactant homeostasis under physiological conditions [35–37, 121]. In lung injury, however, excess LPC uptake through Mfsd2a promoted Drp1-dependent mitochondrial fragmentation, alveolar type 2 cell senescence, and fibrosis in experimental models [36]. The direction of the phenotype therefore depends on substrate load, cellular localization, downstream lipid utilization, and disease stage.

Placental cohorts and models connect MFSD2A to maternal–fetal DHA transfer, gestational diabetes, and fetal growth restriction, but most human findings remain associative [108, 109, 118, 119]. Obesity, age, diet, and metabolic state may accompany altered MFSD2A expression in selected tissues without proving that the transporter causes the systemic disorder. These contexts should be used to define testable transport–phenotype relationships rather than to propose a universal metabolic biomarker.

### Cancer and the tumor microenvironment

Cancer findings should be classified by compartment and study design. Tumor-cell-intrinsic studies support context-specific suppressive associations in lung cancer, colorectal cancer liver metastasis, and hepatocellular carcinoma, but do not define a universal tumor-suppressor program [64, 166, 167]. Tumor-vascular evidence is mechanistically distinct: in brain-metastasis models, reduced endothelial Mfsd2a was linked to BBB leakage, lower DHA transport, and altered lipid metabolism, whereas glioma delivery studies addressed endothelial transcytosis rather than a tumor-cell-autonomous function [161, 163, 168, 169].

Four sources of heterogeneity should therefore be reported explicitly: cellular compartment, substrate environment, downstream lipid utilization, and treatment or disease stage. Tumor-cell expression may influence growth or immune signaling; endothelial expression may primarily regulate transcytosis; circulating-cell expression may reflect host immunity; and bulk-tissue datasets may mix these compartments. In brain tumors, future studies should distinguish tumor core, invasive margin, and uninvolved brain rather than treating vascular permeability and tumor-cell biology as one mechanism.

Cancer-related findings warrant inclusion, but they do not define the manuscript [64, 170, 171]. The relevant evidence comes from distinct settings—advanced gastric cancer, metastatic clear-cell renal cell carcinoma, and lower-grade glioma—with different biospecimens, assays, clinical questions, and levels of mechanistic support [11, 20, 172]. These studies do not establish a pan-cancer pathway, a general prognostic marker, a universal predictor of immunotherapy response, or a causal oncogenic mechanism. Their value lies in defining tumor-specific hypotheses that can be tested without extrapolation.

Gastric cancer and metastatic clear-cell renal cell carcinoma illustrate why direction and context cannot be pooled [173, 174]. In advanced gastric cancer, higher tumor MFSD2A expression has been associated with response to anti-PD-1 therapy; complementary overexpression models linked MFSD2A to changes in T-cell and tumor-associated macrophage populations within the tumor microenvironment [20]. By contrast, patients with metastatic clear-cell renal cell carcinoma showed altered circulating ERVFRD-1 and MFSD2A relative to healthy controls, and progressive disease was associated with higher blood MFSD2A and lower ERVFRD-1 than clinical benefit [11]. These observations arise from different compartments and support no common direction of effect.

Evidence in lower-grade glioma is predominantly correlative. Dataset analyses have linked MFSD2A expression to survival, methylation, immune infiltration, checkpoint genes, angiogenic factors, and BBB-associated transcripts [171, 172, 174, 175]. Such relationships are useful for hypothesis generation, but they do not validate prognosis, treatment selection, or a direct immune mechanism. Table 2 places these findings within an evidence-boundary framework that distinguishes study design, permissible inference, and claims that remain unestablished.

**Table 2:** Evidence tiers, claim boundaries, and translational readiness for MFSD2A-related disease and therapeutic modulation

Domain or proposed claim	Current evidence base	Evidence tier	Permitted interpretation	Claims not established	Translational readiness	Key limitations and unresolved questions	Key references
Na⁺-dependent lysolipid transport by MFSD2A	Cryo-EM structures, mutagenesis, biochemical transport assays, fluorescence-based flippase experiments and tissue-uptake studies	**E1, established**	MFSD2A is a Na⁺-coupled transporter or flippase for LPC-bound fatty acids, with LPC-DHA as the best-characterized cargo	Universal transport of all lipid classes; direct control of every downstream lipid-metabolic or signaling pathway	Established molecular mechanism; suitable for mechanistic assays, but not itself a therapy	Full substrate spectrum, transport stoichiometry, state kinetics, membrane dependence and tissue-specific regulation require further resolution	[2, 21, 22, 128]
MFSD2A-dependent suppression of BBB vesicular transcytosis	Genetic loss-of-function, endothelial models, electron microscopy, tracer permeability, brain lipid measurements and restoration studies	**E1, established**	MFSD2A-dependent lipid transport supports an endothelial membrane state with low caveolae-mediated transcellular transport and low BBB permeability	MFSD2A as the principal tight-junction-closing mechanism; MFSD2A loss as the sole cause of complex barrier failure	Established barrier physiology; no validated clinical intervention	The relative contribution of lipid transport, caveolae, age, disease-specific injury and other endothelial pathways must be quantified in each setting	[47, 82, 123]
Biallelic MFSD2A variants and neurodevelopmental disease	Rare-family genetics, patient plasma-LPC measurements, functional variant studies, endothelial-specific deletion and whole-animal developmental models	**E1, established disease association**	Damaging biallelic variants cause a rare recessive microcephaly-spectrum disorder; impaired transport is plausibly linked to reduced brain lipid delivery, hypomyelination, and abnormal development	A broad neurological biomarker; a uniform genotype–phenotype relationship; a validated diagnostic lipid biomarker or lipid-replacement treatment	Clinical disease recognition and mechanistic modeling; no established disease-modifying therapy	Natural history, residual transporter activity, cell-type contributions, developmental windows, genotype–phenotype correlation and rescue feasibility	[9, 10, 108, 111, 112]
MFSD2A functions outside the BBB	Direct uptake or lipid readouts in retina, lung, kidney, epidermis and T cells; placental cohort, knockdown and developmental evidence	**E2, context-supported**	A shared lysolipid-transport principle is deployed in tissue-specific cellular environments and produces distinct physiological outputs	One universal extra-BBB pathway, identical barrier function across tissues or a common therapeutic window	Context-supported physiology; evidence remains preclinical or observational and tissue specific	Evidence depth is unequal; cell localization, relevant substrates, causal endpoints and compensation require tissue-specific validation	[1, 3, 35, 37, 100]
Acquired MFSD2A dysregulation during barrier injury	Multiple animal and cell models of hypoperfusion, hemorrhage, endothelial signaling loss, infection-related stress, hypoxic-ischemic injury and vascular inflammation	**E2, context-supported**	Reduced or dysregulated MFSD2A can accompany or contribute to loss of the low-transcytosis endothelial state in selected injury settings	A universal initiating mechanism for all BBB disorders; a single linear upstream pathway; equivalence of all injury models	Preclinical disease biology	Marker-versus-mediator status, temporal order, disease-specific triggers, transcellular versus paracellular contributions and reversibility	[7, 53, 55, 136, 138]
Barrier-protective direction through MFSD2A preservation or restoration	Overexpression or intervention studies in selected hypoperfusion, hemorrhage, traumatic-injury and vascular-stress models with permeability and vesicle readouts	**E2, context-supported preclinical concept**	Restoring MFSD2A-associated restraint can reduce vesicular transcytosis and barrier leakage in defined models	A validated MFSD2A activator; a general clinical barrier-protection strategy; safety across retina, placenta and other endothelial beds	Exploratory preclinical concept	Selective on-target pharmacology, dose window, timing, regional specificity, durability and off-target barrier effects	[7, 53, 61]
Transient MFSD2A attenuation to increase CNS or tumor-barrier delivery	Wnt/MFSD2A manipulation in glioma models, nanoparticle delivery studies in brain metastasis and in vitro BBB systems	**E2, context-supported preclinical concept**	Attenuating the MFSD2A-low-transcytosis program can increase cargo exposure in selected experimental settings	Healthy-BBB sparing, selective blood-tumor barrier opening, universal cargo benefit, reversibility or clinical feasibility	Exploratory preclinical concept with a high safety burden	Regional and endothelial-bed specificity, cargo dependence, duration, nonselective plasma-protein entry, neurotoxicity and recovery of barrier function	[141, 148, 149]
LPC-DHA carrier or dietary lipid supplementation	Dietary, carrier-formulation, retinal, placental and brain-lipid studies with variable transporter and tissue endpoints	**E3, exploratory**	Substrate form and availability can influence tissue DHA delivery when transporter capacity, timing and tissue demand are favorable	Correction of MFSD2A loss-of-function disease; a general recommendation to increase PUFA intake; equivalence between substrate supply and MFSD2A modulation	Exploratory nutritional and preclinical concept	Transporter dependence, formulation, dose, developmental timing, tissue access, compensatory metabolism and controlled human outcomes	[5, 88, 89, 94]
Selective pharmacological activation or inhibition of MFSD2A	Predominantly indirect pathway interventions, delivery concepts and review-level proposals; no mature selective modulator is established in the manuscript evidence base	**Boundary-only**	Development of selective MFSD2A modulators is a future experimental objective requiring direct target-engagement and transport assays	An existing clinically validated activator, inhibitor or MFSD2A-directed treatment platform	Not ready	Molecular selectivity, on-target confirmation, pharmacokinetics, tissue distribution, bidirectional barrier effects, reversibility and systemic safety	[141, 148, 149]
Gastric cancer and anti-PD-1-associated observations	A selected clinical cohort plus complementary tumor-cell overexpression and preclinical immune-microenvironment readouts	**E2, context-supported**	In the reported gastric-cancer setting, higher tumor MFSD2A was associated with anti-PD-1 response, and experimental elevation of MFSD2A was linked to remodeling of the tumor microenvironment	A universal gastric cancer biomarker, pan-cancer immunotherapy predictor or validated MFSD2A-directed therapy	Exploratory tumor-specific biomarker and mechanistic hypothesis	Independent replication, prospective design, assay standardization, cutoff definition, causal requirement for transport activity and external validation	[20]
Metastatic clear-cell renal cell carcinoma circulating association	Small peripheral-blood cohort measuring ERVFRD-1 and MFSD2A expression in relation to clinical benefit or progressive disease	**E3, associative**	A pilot blood-based association can be reported in its specific cohort and biospecimen context	The cellular source of the signal, tumor-tissue function, a causal immune mechanism or a validated predictive biomarker	Observational only	Sample size, source-cell identity, treatment and clinical confounding, assay reproducibility and prospective validation	[11]
Lower-grade glioma expression, survival and immune associations	Retrospective transcriptomic and clinical-dataset analyses linking MFSD2A expression with survival, methylation and immune-associated features	**E3, associative**	Hypothesis-generating, dataset-level associations in lower-grade glioma	Causality, a direct immune-regulatory mechanism, prospective prognostic use, treatment selection or therapeutic targeting	Observational only	Dataset dependence, tumor heterogeneity, cell-of-origin ambiguity, technical validation and prospective cohort confirmation	[158]
Direct MFSD2A-ACSL4/LPCAT3-ferroptosis mechanism	No direct same-system causal chain linking MFSD2A transport, ACSL4/LPCAT3 engagement, defined lipid species and ferroptotic death is established in the accepted evidence base	**Boundary-only**	This can be named only as an unresolved hypothesis requiring direct transport, enzyme, lipidomic, rescue and cell-death experiments	MFSD2A as an established ferroptosis regulator or a direct ACSL4/LPCAT3 pathway input	Not ready	Transport-dead controls, substrate-defined rescue, enzyme perturbation, lipid-species resolution and exclusion of non-ferroptotic death	[21, 22, 39]
MFSD2A as a pan-cancer biomarker or universal prognostic factor	Tumor studies differ in cancer type, biospecimen, assay, direction of association and evidentiary depth; the original broad cancer catalogue exceeds the revised manuscript's supported scope	**Boundary-only**	MFSD2A-related observations should be reported separately by tumor type, specimen and study design	A unified pan-cancer direction of effect, universal prognosis marker or broad immunotherapy-response biomarker	Not ready	Prospective tumor-specific cohorts, harmonized assays, predefined endpoints, independent replication and mechanistic validation	[11, 20, 158]
Universal MFSD2A loss across BBB disorders	Repeated MFSD2A reduction is reported in several injury models, but initiating insults, endothelial programs and permeability routes are heterogeneous	**Boundary-only**	Recurrent context-specific dysregulation can be described, with each disease model and causal position stated separately	MFSD2A loss as the universal origin, necessary event or sufficient explanation for BBB dysfunction	Not a clinical claim	Temporal causality, marker-versus-mediator status, model comparability, disease specificity and interaction with junctional, inflammatory and hemodynamic injury	[7, 53, 55, 136]

This table separates biological evidence tiers from translational readiness and identifies claims that remain unsupported

Evidence tier and translational readiness are considered separately: an E1 or E2 biological relationship may still lack a validated therapeutic application. ‘Not ready’ indicates that current evidence does not support clinical action or a mature translational strategy

**Fig. 6:**
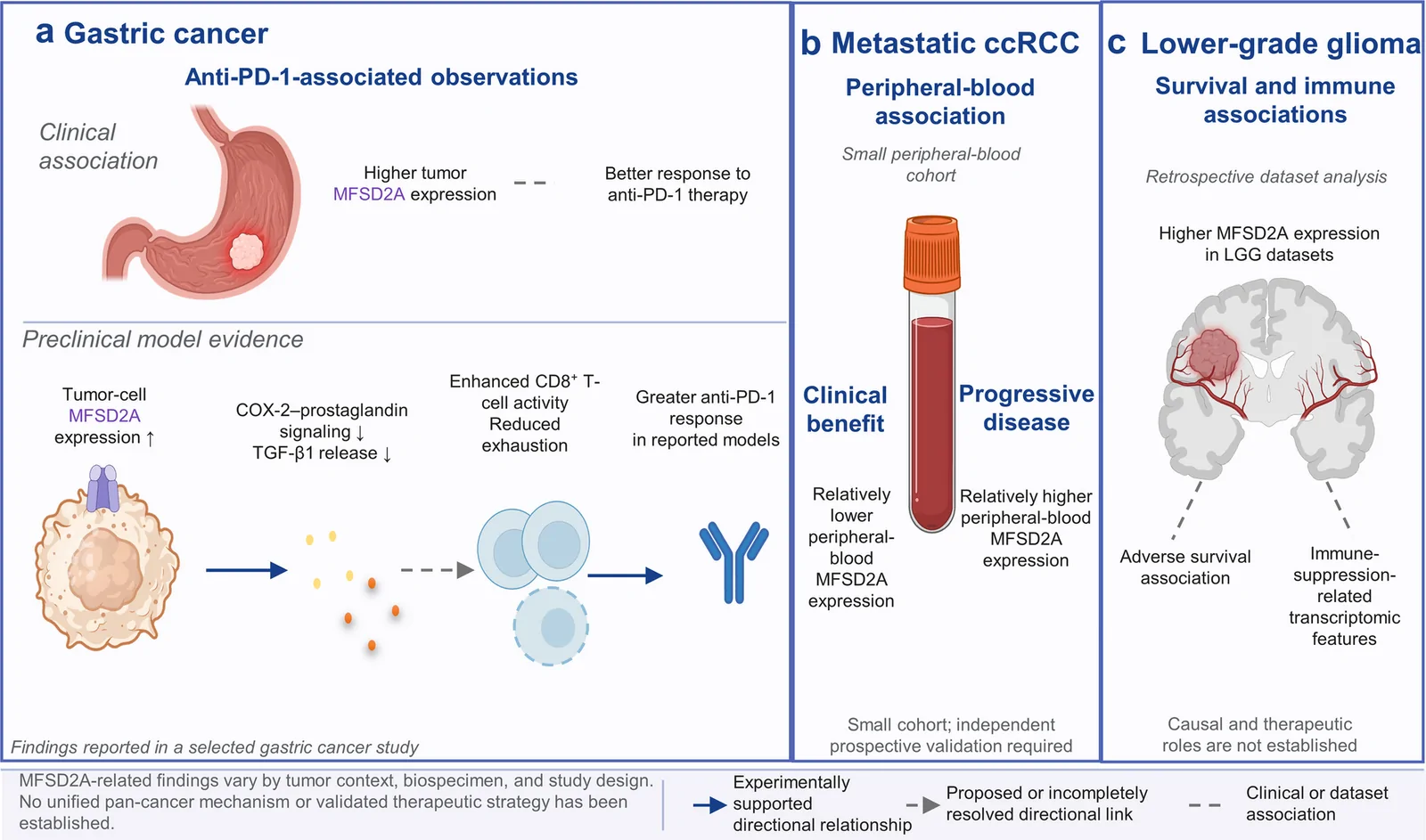
Selected tumor-context and immunotherapy-associated observations involving MFSD2A. **a** Gastric cancer and anti-PD-1 treatment. In one clinical cohort, higher tumor MFSD2A expression was associated with a better response to anti-PD-1 therapy. Complementary preclinical experiments linked tumor-cell MFSD2A overexpression to reduced COX-2–prostaglandin signaling and TGF-β1 release, greater CD8⁺ T-cell activity, less T-cell exhaustion, and enhanced anti-PD-1 responsiveness. This combination of clinical association and experimental evidence is study specific and requires independent validation. **b** Metastatic clear-cell renal cell carcinoma. In a small cohort, patients with clinical benefit had lower peripheral-blood MFSD2A expression than those with progressive disease. The cellular source, relationship to tumor-tissue expression, and predictive value remain unknown. **c** Lower-grade glioma. Retrospective datasets associated higher MFSD2A expression with adverse survival and immunosuppression-related transcriptional features, but do not establish causality, direct immune regulation, or therapeutic relevance. Solid arrows indicate experimentally supported directional relationships; gray dashed arrows indicate proposed or incompletely resolved links; dashed lines without arrowheads indicate clinical or dataset associations. Across the three settings, the direction and meaning of MFSD2A-related findings depend on tumor type, biospecimen, and study design. No unified pan-cancer mechanism, universal biomarker, or validated MFSD2A-directed therapy has been established. ccRCC, clear-cell renal cell carcinoma; COX-2, cyclooxygenase-2; LGG, lower-grade glioma; PD-1, programmed cell death protein 1; TGF-β1, transforming growth factor-β1

Immunotherapy-related language requires particular restraint [176]. MFSD2A-dependent LPC uptake in activated T cells establishes immune-cell transport biology, and the gastric cancer study supports a tumor-specific relationship with the immune microenvironment [3, 20]. Neither finding demonstrates that MFSD2A reverses T-cell exhaustion or predicts anti-PD-1 response across malignancies. Likewise, blood-based renal cell carcinoma data and glioma transcriptomic correlations cannot be combined into a pan-cancer biomarker [11, 172]. Oncology consequently remains a downstream, tumor-specific domain for validation rather than an actionable application of MFSD2A biology. Figure 6 compares the compartment, evidence type, and permitted interpretation across the three selected tumor settings.

Maintaining this tumor-specific classification is not merely taxonomic. Gastric cancer, metastatic clear-cell renal cell carcinoma, and lower-grade glioma differ in tissue of origin, biospecimen, immune environment, vascular context, and assay design [11, 20, 172]. Grouping them under a generic gastrointestinal, hematological, or pan-cancer label would erase the very features needed for interpretation. These studies should instead be viewed as bounded examples that justify further investigation while withholding prognostic, predictive, and therapeutic claims pending prospective validation [177].

## Translational opportunities and therapeutic constraints

This section evaluates translation in two linked but distinct domains: deliberate modulation of endothelial permeability and the development of human biomarkers, patient-stratification approaches, and drug-discovery tools. Each is assessed against the available evidence for selectivity, reversibility, tissue specificity, safety, and prospective validation.

### Barrier modulation and drug delivery: translational potential and unresolved safety issues

The barrier literature raises a focused translational question: whether the endothelial state governed in part by MFSD2A can be manipulated to protect the BBB or control CNS exposure. The biological rationale is strongest at the BBB, where Mfsd2a supports barrier maturation, suppresses endothelial transcytosis, and couples DHA-dependent membrane composition to low vesicular traffic [6–8]. This makes MFSD2A relevant to delivery research, but not a validated delivery platform. The appropriate benchmark is experimental feasibility rather than preclinical or clinical readiness [178].

Intervention evidence remains much weaker than the underlying barrier biology. Studies in CNS and sensory barriers have linked Mfsd2a expression or inhibition to transcytosis, barrier maturation, blood-retinal and blood-labyrinth permeability, focused-ultrasound-associated transport windows, and properties of in vitro BBB models [68, 125, 179–181]. These observations identify settings in which the MFSD2A endothelial state may be experimentally tractable. Most are specific to a particular model, timing, cargo, or vascular bed, and collectively support a research program in permeability control rather than a claim of improved CNS drug delivery.

Safety and selectivity remain unresolved, particularly when BBB mechanisms are extrapolated to the blood-tumor barrier [182]. Altering vesicular transport in one endothelial bed may compromise physiological restriction elsewhere [183]. Current studies do not show that healthy BBB can be spared, that tumor and non-tumor vessels respond differently enough to create a useful window, or that any increase in permeability would be regional, reversible, and cargo selective [7, 180, 181]. The Wnt/MFSD2A/BBB axis captures this tension: a mechanism that protects barrier integrity may oppose attempts to enhance delivery. That conflict must be tested rather than assumed away.

Three distinct translational concepts should be kept separate. The first is preservation of MFSD2A function during injury to stabilize the barrier [6–8]. The second is transient modulation of transcytosis to increase delivery of a defined cargo [168, 169, 180]. The third is alteration of substrate supply to change tissue lipid composition. Each has different endpoints, risks, and safety requirements [184]. Protecting the barrier is not equivalent to opening it, and increasing lipid availability is not equivalent to manipulating vesicular traffic. Conflating these approaches would turn a physiological mechanism into a premature delivery claim [185].

The blood-tumor barrier requires an additional layer of caution [186]. Tumor-associated vessels differ from normal CNS endothelium in permeability, signaling, cellular support, immune context, and regional heterogeneity [187]. The fact that MFSD2A maintains physiological BBB restriction does not imply that its inhibition in tumor vessels would selectively enhance drug delivery while sparing healthy brain [7, 180, 181]. A more tractable question is whether tumor-associated endothelial states create a measurable and reversible difference in MFSD2A-dependent vesicular restraint. The problem remains one of safety and feasibility rather than an established translational opportunity.

Future studies should use endpoints proportionate to the risk. Reversibility, regional specificity, dose window, cargo dependence, endothelial-bed selectivity, separation of blood-tumor and healthy BBB effects, and off-target barrier injury all require direct measurement [6, 7, 180, 181]. Experiments must also distinguish suppression of transcytosis, induction of transcytosis, and modification of lipid substrate supply, because these interventions are mechanistically and clinically different. Until such criteria are met, MFSD2A is best regarded as a biologically plausible node for barrier modulation with unresolved safety constraints [188].

### Human biomarker evidence, patient stratification, and clinical-trial landscape

Human evidence spans distinct levels and should not be interpreted as a single translational trajectory. Biallelic pathogenic variants provide a causal basis for the molecular diagnosis of MFSD2A-associated microcephaly [9, 130], but these genetic studies do not establish MFSD2A as a quantitative circulating biomarker. In oncology, a small hepatocellular-carcinoma study reported lower plasma MFSD2A in 21 patients than in hepatitis-B and healthy control groups, with moderate discrimination (area under the receiver operating characteristic curve, 0.718); tissue expression in a separate cohort of 79 patients was also associated with survival. Retrospective analyses of public lower-grade-glioma datasets similarly associated MFSD2A expression with survival and immune-related transcriptomic features [171, 172]. These findings support exploratory diagnostic or prognostic hypotheses, not clinically validated biomarker applications.

Evidence relevant to treatment stratification is also preliminary and context dependent. In advanced gastric cancer, higher tumor MFSD2A expression was associated with a better response to anti-PD-1 therapy among 27 evaluable patients, whereas a pilot study of 34 patients with metastatic clear-cell renal-cell carcinoma associated higher pretreatment peripheral-blood MFSD2A expression with progressive disease rather than clinical benefit [11, 20]. The different biospecimens, tumor types, and directions of association preclude a universal threshold or cross-cancer interpretation. MFSD2A should therefore be regarded as a candidate component of future stratification models rather than an established companion diagnostic or precision-medicine marker. Prospective studies would require prespecified assays and cutoffs, compartment-resolved sampling, adjustment for established clinicopathological predictors, and independent validation before clinical use.

Near-term human studies should pair MFSD2A measurements with functional endpoints. Rare-disease studies require variant-specific surface expression, sodium-coupled LPC flux, lipidomics, neuroimaging, and longitudinal development; barrier studies require permeability measurements aligned with disease stage; and cancer studies require spatial resolution of malignant, endothelial, and immune compartments together with treatment exposure and LPC species. Such designs can distinguish a causal mediator or pharmacodynamic marker from a correlated feature of cellular state.

ClinicalTrials.gov record NCT06071598 is a small, single-center, cross-sectional observational study of colorectal-cancer surgical specimens and MFSD2A-related inflammatory-resolution biology. It is not a drug-treatment trial of an MFSD2A-selective agonist or inhibitor; its status is currently listed as unknown, with no results posted [189]. As of July 2026, no clinically approved MFSD2A-selective agonist or inhibitor and no completed interventional trial demonstrating therapeutic benefit from direct MFSD2A modulation were identified.

Current structural and functional studies provide tools that could enable drug discovery rather than an established MFSD2A drug-discovery platform. Cryo-electron-microscopy structures define substrate- and sodium-binding regions, while cell-based lysolipid-uptake and reconstituted-proteoliposome flippase assays provide functional readouts for candidate modulators [21, 22]. However, these methods have not yet yielded a clinically validated selective modulator or a demonstrated therapeutic window. Cross-species microvessel proteomics and BBB-delivery literature also caution that assay activity and cargo exposure may not predict native human vascular responses [46, 190–192]. Clinical readiness should be assigned separately from biological evidence strength. Translation therefore requires assay qualification, selective pharmacological probes, target-engagement measurements, tissue-specific delivery, and direct evaluation of reversible barrier and lipid-transport effects.

## Controversies and future perspectives

MFSD2A is most firmly positioned at the intersection of sodium-dependent lysolipid transport, endothelial barrier specialization, and rare neurodevelopmental disease. Established observations include transport of LPC-bound substrates, impaired brain and retinal DHA delivery after loss of function, suppression of vesicular transcytosis at CNS barriers, pathogenic biallelic variants in microcephaly syndromes, and experimental links between endothelial transport and brain growth [2, 6, 10]. The major questions now concern causal depth, tissue specificity, and whether any aspect of this biology can be manipulated safely.

### Established mechanisms and unresolved claims

The first unresolved issue is how far transport biology can be extended downstream [65]. MFSD2A-mediated LPC and LPC-DHA transport is linked to endothelial lipid composition, caveolar restraint, and brain DHA availability [2, 8, 10, 103]. It does not follow that every pathway involving phospholipid remodeling is directly controlled by MFSD2A. In particular, ACSL4/LPCAT3 coupling and ferroptosis remain unproven. Testing these ideas will require transporter activity, defined lipid species, enzyme engagement, and phenotype to be measured within the same experimental system.

A second question is whether endothelial membrane effects extend to cancer-cell lipid rafts. Direct evidence currently supports lysolipid transport, endothelial membrane state, and caveolar suppression [2, 8]. MFSD2A-dependent lipid-raft remodeling in tumor cells has not been demonstrated. The tractable experiment is to test whether altered substrate transport changes membrane composition or signaling in a defined cancer type under controlled conditions [20, 64, 166, 167]. Until then, lipid-raft models should remain explicitly speculative.

A third issue concerns substrate supplementation and pharmacological activation. Maternal–fetal and dietary DHA studies motivate questions about lipid supply, while tumor studies raise separate questions about immune context [20, 108, 109]. These lines of evidence do not support a combined strategy in which MFSD2A activation and dietary PUFA sensitize immunologically cold tumors. Such a proposal requires direct intervention, tumor-specific efficacy, immune readouts, pharmacodynamic evidence, and safety testing before it can move beyond hypothesis.

Barrier modulation creates the fourth and most immediate translational controversy. The same MFSD2A biology that makes permeability experimentally interesting also creates the safety risk. Increasing or decreasing transcytosis could affect the healthy BBB, retina, blood-labyrinth barrier, placenta, and other vascular interfaces. Current studies do not demonstrate healthy-BBB sparing, absence of off-target barrier injury, or selective separation of tumor and non-tumor endothelium [7, 180, 181]. Safety remains an open experimental question, not an assumption that can be embedded in a delivery proposal.

The fifth controversy is the interpretation of oncology data. Advanced gastric cancer, metastatic clear-cell renal cell carcinoma, and lower-grade glioma represent distinct biological and clinical settings. Gastric cancer data support a study-specific association with anti-PD-1 response and immune-microenvironment remodeling [20]. Renal cell carcinoma data describe a circulating-marker association [11]. Glioma evidence is primarily retrospective and hypothesis generating [172]. Prospective cohorts, standardized assays, and tumor-specific mechanistic studies are required before any MFSD2A measurement can be considered clinically actionable.

These uncertainties become productive only when evidence levels remain visible. Transporter and BBB mechanisms provide the anchor [2, 6, 10]. Downstream lipid models require causal testing; barrier interventions require evidence of specificity and reversibility; and cancer associations require validation within the same tumor type and biospecimen [20]. The strength of the MFSD2A field lies in a concrete molecular mechanism, but its broader implications depend on maintaining the distinction between established causality, model-specific association, and testable hypothesis.

Breadth should be prioritized by evidentiary depth rather than by the number of reported associations. The most coherent progression runs from structure and transport to CNS barrier physiology, tissue-specific lipid handling, neurodevelopmental disease, acquired barrier dysfunction, and only then to restricted translational contexts [2, 6, 10, 11]. This ordering preserves the mechanistic center while showing readers what is established, what remains plausible, and what should remain outside the main narrative. Table 2 summarizes the resulting evidence tiers, claim boundaries, and translational readiness.

### Priorities for causal biology, safety, and translation

The next phase of MFSD2A research should deepen causality rather than expand the list of associations. Mechanistic studies need to connect transporter structure, sodium coupling, substrate selectivity, lipid species, membrane composition, vesicular traffic, and tissue phenotype within the same system [2, 10, 21, 22]. This is particularly important for proposed effects beyond LPC transport. Experimental designs that separate transporter activity from substrate availability, endothelial maturation, injury signaling, and compensatory metabolism will identify which consequences are intrinsic to MFSD2A and which arise from tissue context.

Barrier-focused work should determine when MFSD2A functions as a developmental requirement, a maintenance factor in mature endothelium, or a stress-responsive component of injury. Time-resolved studies should integrate endothelial maturation, pericyte support, Wnt-linked signaling, vascular inflammation, caveolar dynamics, and direct lipid measurements [44, 45, 78, 79]. The key objective is to distinguish regulation of MFSD2A itself from broader changes in endothelial identity that merely correlate with its expression.

Clinical genetics requires better genotype–phenotype resolution. Additional patient series, standardized clinical phenotyping, plasma and tissue lipid measurements, and functional analysis of individual variants would refine the spectrum of MFSD2A-associated microcephaly [9, 128, 131, 132]. Models should test how residual transport, timing of loss, endothelial specificity, and neural or glial lipid demand influence severity [10]. Such studies could sharpen disease mechanism and identify realistic rescue windows without implying an unproven treatment.

Translational studies should meet the same causal standard. Barrier modulation requires direct evidence of reversibility, regional specificity, cargo dependence, dose window, vascular-bed selectivity, and off-target barrier effects [180, 181]. Oncology studies require prospective, tumor-specific validation before MFSD2A-related measurements move beyond descriptive association [11, 20]. Progress will depend on extending the transport-to-disease framework without converting biological plausibility into practice-facing claims.

The most productive path is to reinforce this structure rather than widen the claims indiscriminately. Transporter mechanism, BBB transcytosis, developmental genetics, tissue-specific lipid handling, and tumor-context validation each require deeper causal resolution [2, 6, 10, 20]. None depends on importing unsupported therapeutic narratives. Keeping structure, physiology, tissue context, genotype–phenotype relationships, translational opportunity, safety, and uncertainty analytically distinct will allow the literature to grow without outrunning its evidence.

MFSD2A is a useful model for disciplined, transporter-centered disease biology. A well-defined transport mechanism can make downstream disease links biologically plausible, but plausibility is not equivalent to causality, safety, or clinical utility. The best-supported sequence runs from sodium-dependent LPC transport to endothelial lipid state, barrier physiology, and neurodevelopmental phenotype [2, 6, 10]. Drug-delivery and oncology applications remain informative only when tissue specificity, safety uncertainty, and mechanistic limits are preserved [11]. This balance provides a durable framework for future work while keeping the central claims auditable. Across systems, the most informative explanatory variables are tissue and cellular localization, substrate identity and abundance, downstream lipid utilization, and disease stage. Keeping these variables explicit provides a testable framework for apparently divergent outcomes without assuming a universal dual-role mechanism.

## Data Availability

No datasets were generated or analysed during the current review. All information discussed is available in the cited publications and public study records.
